# Perceived worries in the adoption of artificial intelligence among nurses in neonatal intensive care units

**DOI:** 10.1186/s12912-025-03318-z

**Published:** 2025-07-01

**Authors:** Ahmad Ayed, Ahmad Batran, Ibrahim Aqtam, Malakeh Z. Malak, Moath Abu Ejheisheh, Mosaab Farajallah, Lamees Farraj, Sanaa Alkhatib

**Affiliations:** 1 Faculty of Nursing, Arab American University, Jenin, Palestine; 2 Faculty of Allied Medical Sciences, Department of Nursing, Palestine Ahliya University, Bethlehem, Palestine; 3 Department of Nursing, Ibn Sina College for Health Professions, Nablus University for Vocational and Technical Education, Nablus, Palestine; 4 Community Health Nursing, Faculty of Nursing, Al-Zaytoonah University of Jordan, Amman, Jordan

**Keywords:** Artificial intelligence, Technology adoption, Healthcare, Neonatal intensive care, Nurses

## Abstract

**Introduction:**

Artificial Intelligence (AI) comprises computational algorithms designed to analyze data, learn patterns, and execute tasks traditionally requiring human cognition. These models can support public health initiatives, expedite clinical care, and improve diagnosis accuracy. Thus, artificial intelligence in healthcare sectors has the potential to enhance nursing care by assisting nurses with tasks like documentation, workflow improvement, and decision-making, while reducing workforce stress. This study, guided by the Technology Acceptance Model (TAM), assesses perceived worries regarding AI adoption among nurses in neonatal intensive care units (NICUs).

**Methods:**

A cross-sectional quantitative design was employed using convenience sampling. Data were collected using the Worries of Applying AI in Healthcare Questionnaire (WAAI-HCQ) from 227 NICU nurses across nine hospitals in the West Bank (January 2–March 3, 2025). SPSS version 26 was used for analysis.

**Results:**

Participants demonstrated intermediate levels of AI awareness (M = 2.7, SD = 0.5) and limited prior AI experience (M = 2.3, SD = 0.5). Total AI-related worries were moderate (M = 3.2, SD = 0.9), with healthcare provider-related concerns being highest. Multiple linear regression (R² = 0.846) identified education level (B = 0.074, *p* = 0.026), AI awareness (B = 2.006, *p* < 0.001), and AI experience (B = -0.959, *p* < 0.001) as significant predictors, explaining 84.6% of the variance in AI-related worries.

**Conclusions:**

NICU nurses in Palestine exhibit moderate AI awareness and concerns, highlighting the need for targeted education and training to address knowledge gaps and facilitate AI integration. This study contributes new knowledge specifically for conflict-affected, resource-constrained NICU settings, where AI implementation faces unique challenges.

**Clinical trial number:**

Not applicable.

## Introduction

Artificial Intelligence (AI) encompasses computational systems designed to perform tasks requiring human-like intelligence, with transformative potential in healthcare. In nursing, AI applications promise to streamline documentation, enhance decision-making, and reduce clinical burdens; however, their integration raises specific AI worries, defined here as nurses’ perceived risks and concerns regarding the implementation of AI technologies in healthcare settings, including ethical dilemmas, role displacement, and workflow disruptions, distinct from broader concerns about technical feasibility or acceptance [1, 2].

This study is grounded in the Technology Acceptance Model (TAM), which provides a structured theoretical framework for understanding technology adoption in organizational contexts. TAM emphasizes *perceived usefulness* (the degree to which users believe a technology will enhance their job performance) and *perceived ease of use* (the degree to which users believe using a technology will be free of effort) as primary determinants of technology adoption [2]. In healthcare contexts, these constructs interact with professional concerns and contextual factors to influence acceptance.

### Conceptual framework

**Fig. 1:**
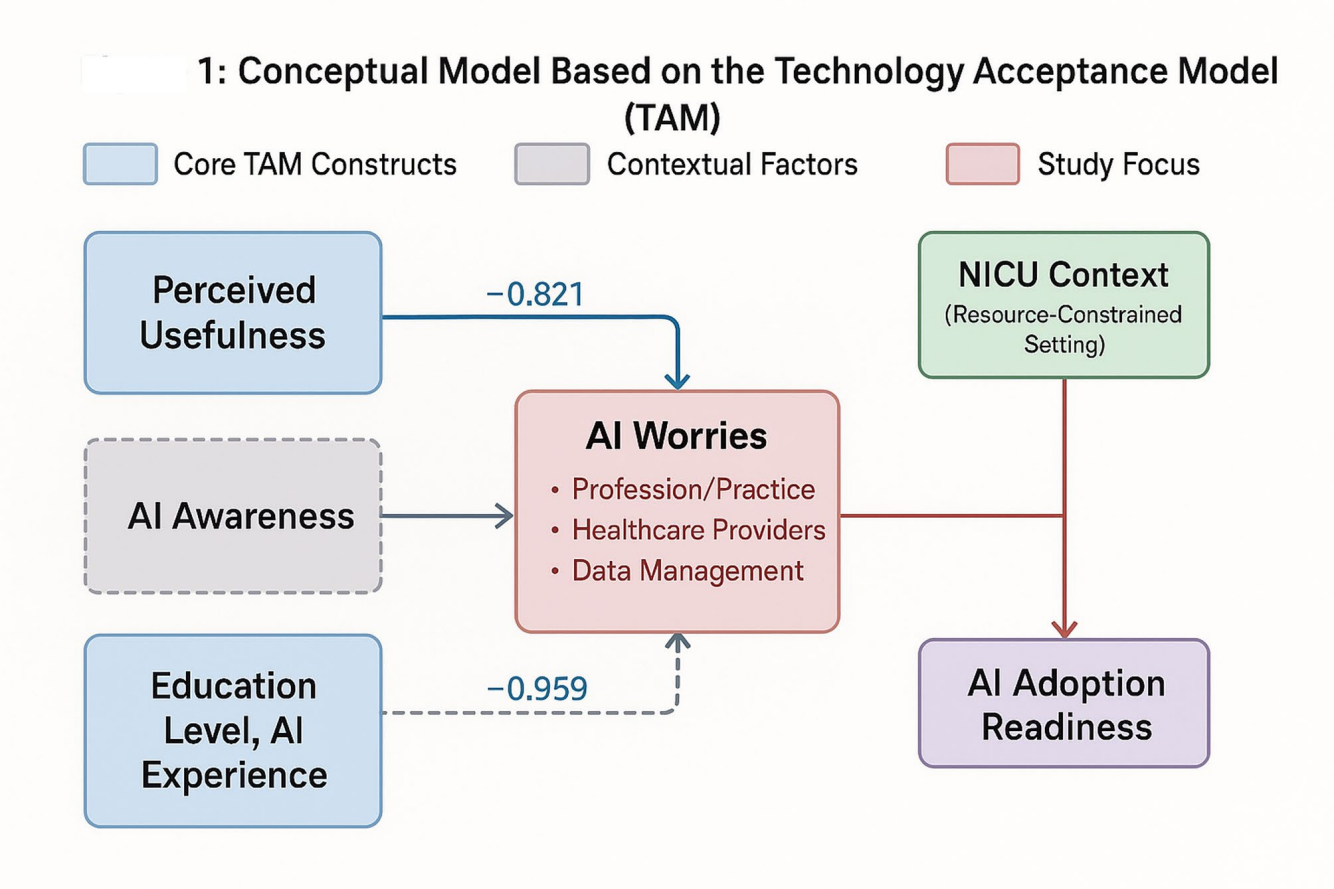
Technology Acceptance Model (TAM)-Based Conceptual Framework for AI-Related Worries in NICU Nurses. This framework demonstrates how TAM constructs (perceived usefulness and ease of use) interact with contextual factors specific to Palestinian NICUs to influence AI-related worries. The model shows that while perceived usefulness may drive acceptance, infrastructural barriers and professional concerns can heighten worries despite recognition of AI benefits. Arrows indicate hypothesized relationships, with solid lines representing direct TAM relationships and dashed lines representing contextual modifications relevant to conflict-affected, resource-constrained settings

Our conceptual framework illustrates how TAM constructs relate to AI-related worries in Palestinian NICUs, where unique infrastructural constraints such as intermittent power supply, limited internet connectivity, and resource scarcity may significantly influence perceptions of both usefulness and ease of use (Fig. 1). The framework demonstrates that while perceived usefulness may drive acceptance, infrastructural barriers and professional concerns can heighten worries despite recognition of AI benefits. This adaptation of TAM to low-resource, conflict-affected settings extends the model’s applicability beyond traditional Western healthcare contexts.

### Components of the framework

- **Perceived Usefulness**: Recognition of AI’s potential to improve clinical decision-making, reduce documentation burden, and enhance patient outcomes.
- **Perceived Ease of Use**: Concerns about implementation complexity given infrastructure limitations and training requirements.
- **Contextual Factors**: Infrastructure constraints (power outages, connectivity issues), resource limitations, and conflict-related challenges.
- **Professional Concerns**: Role displacement fears, skill obsolescence, and ethical considerations.
- **AI-Related Worries**: The outcome variable representing nurses’ comprehensive concerns about AI implementation.

While global research explores AI adoption in healthcare [3, 4], neonatal care remains underexamined. NICU nurses manage critically ill infants requiring rapid, precise interventions, making this setting uniquely sensitive to technological disruptions. AI tools, such as predictive analytics for neonatal sepsis or automated vital monitoring, could reshape workflows, yet their impact on nursing roles in resource-constrained regions like Palestine remains unstudied. Existing literature focuses on Western contexts or general nursing populations [5, 6], leaving gaps in understanding how cultural, infrastructural, and clinical factors in low-resource settings shape AI-related concerns.

In Palestine specifically, healthcare delivery occurs within a complex geopolitical context characterized by movement restrictions, intermittent access to advanced technologies due to import limitations, and fragmented healthcare infrastructure. These unique challenges create a distinct environment where traditional TAM assumptions about technology accessibility and organizational support may not be held, particularly given the ethical considerations and implementation challenges associated with AI tools in healthcare settings [7]. Palestinian NICU nurses operate under conditions where equipment maintenance is challenging, power outages are frequent, and access to technological training is limited. These factors necessitate a contextualized understanding of how AI adoption concerns manifest differently from high-resource settings.

This study addresses these gaps by investigating Palestinian NICU nurses’ awareness, experience, and perceived worries regarding AI. By situating the analysis within TAM, we aim to elucidate how cognitive and contextual factors influence readiness for AI integration, offering insights for tailored training and policy in neonatal care.

## Methods

### Design and settings

This cross-sectional study examined AI-related worries among NICU nurses in Palestine using the Worries of Applying AI in Healthcare Questionnaire (WAAI-HCQ). The questionnaire was administered in its original English version, as English is the primary language of nursing education and clinical practice in Palestine. To ensure cultural relevance, minor adaptations were made during pilot testing with 20 NICU nurses (e.g., replacing ‘HIPAA compliance’ with ‘local data privacy norms’).

### Instrument validation

The WAAI-HCQ demonstrated strong psychometric properties in the pilot study. Internal consistency was confirmed across all subscales (Cronbach’s α = 0.85–0.92), and construct validity was supported by significant correlations among subscales. Test-retest reliability over a two-week interval showed stable correlations (*r* = 0.78–0.85). However, confirmatory factor analysis (CFA) was not conducted due to sample size constraints (pilot *n* = 20, insufficient for CFA requirements). While the WAAI-HCQ has been previously validated in similar Middle Eastern populations [5], future validation studies with larger samples (*n* ≥ 200) are explicitly recommended to confirm the factor structure of the WAAI-HCQ specifically in Palestinian healthcare settings and establish measurement invariance across different cultural contexts.

Data collection occurred from January 2 to March 3, 2025, across nine hospitals (three governmental, three non-governmental, three university) in the West Bank.

### Sampling and limitations

A convenience sample of 227 nurses was recruited from nine NICUs. Sample size was calculated using Raosoft software (population = 355, margin of error = 5%, confidence level = 95%, response distribution = 50%) [8], yielding a minimum target of 185. To account for a 27% anticipated attrition rate, common in hospital-based surveys [9], 235 nurses were initially enrolled.

### Critical sampling limitations

This convenience sampling approach represents a significant methodological limitation with several important implications for the generalizability and interpretation of our findings:

- **Systematic Exclusion Bias**: Convenience sampling may have introduced systematic exclusion bias by omitting key subgroups of NICU nurses. Nurses working in smaller or remote facilities with limited research exposure may not have been represented. Night-shift nurses, who often have different patterns of technology use, could also have been missed. The sample may have excluded nurses with low technological engagement who are less likely to participate in studies involving digital tools. Part-time or contract nurses, who often experience different levels of organizational integration, might also be underrepresented.
- **Potential Overrepresentation**: This sampling bias could have resulted in an overrepresentation of more technologically aware and engaged nurses, potentially inflating AI awareness scores and creating a sample that may not fully represent the broader Palestinian NICU nurse population’s true technology readiness levels.
- **Geographic Limitations**: The exclusion of nurses from Gaza due to access restrictions further limits generalizability to the entire Palestinian nursing workforce, representing approximately 30% of the total Palestinian nurse population.

### Non-responder analysis limitations

The lack of non-responder analysis is a critical limitation. Institutional policies and privacy restrictions prevented access to demographic data for nurses who declined participation. This made it impossible to compare responders and non-responders on key characteristics such as age, attitudes toward technology across generations, years of nursing experience, adaptability to change, educational background, engagement in continuing education, and prior exposure to technology initiatives. This gap raises concerns about response bias. Nurses who chose to participate may have differed systematically from those who declined, especially in their interest in technology, openness to innovation, or confidence discussing technology-related topics. These differences could skew the findings related to AI-related concerns and limit how well the results represent the broader NICU nursing population.

### Inclusion criteria

Full-time NICU nurses with > 1 year of experience and proficiency in English (confirmed via self-assessment during recruitment) were included.

### Data analysis

Data were analyzed using SPSS v26 [10]. Double-entry verification and random checks ensured accuracy. Descriptive statistics summarize demographics and AI worries. Pearson/Spearman correlations assessed relationships between variables. Multiple regression assumptions (normality, homoscedasticity, multicollinearity) were validated using Shapiro-Wilk tests [11], residual plots, and variance inflation factors (VIFs < 2.0).

### Addressing the high R² value

The exceptionally high R² value (0.846) observed in this study requires comprehensive interpretation, as such values are uncommon in social science research and warrant careful methodological consideration:

### Potential explanations for high explained variance

- **Strong Predictor Intercorrelations**: The remarkably strong correlation between AI awareness and AI worries (*r* = 0.821) suggests these constructs may be more interrelated in Palestinian healthcare settings than in other contexts. This relationship may reflect the reality that in environments with limited AI exposure, increased awareness primarily comes through exposure to challenges and limitations rather than benefits, creating a stronger awareness-worry link than might exist in settings with more diverse AI experiences.
- **Sample Homogeneity Effects**: The sample’s relative homogeneity may have reduced variation in responses and limited the study’s ability to detect nuanced patterns. All participating hospitals faced similar infrastructural constraints, which likely shaped nurses’ work experiences in consistent ways. Participants also appeared to share cultural backgrounds and professional socialization experiences, reducing diversity in values and attitudes. Organizational contexts were largely comparable, with similar resource limitations that could affect technology adoption. Prior exposure to AI also showed limited variation, further narrowing the range of perspectives captured. This homogeneity may have masked important subgroup differences and constrained the generalizability of findings.
- **Contextual Uniformity**: The unique characteristics of Palestinian healthcare may have created more uniform response patterns compared to populations with diverse healthcare infrastructure and AI exposure levels.

### Methodological considerations

Bootstrap validation using 1,000 samples was used to reduce overfitting risk and confirmed model stability, with a bootstrap R² range of 0.821 to 0.859. Despite this, several limitations remain. The high explained variance may not generalize to more diverse populations or other cultural settings. Unmeasured confounding variables specific to the Palestinian healthcare context, such as organizational culture, political instability, and distinctive professional socialization, may have influenced the results. The model may also be overfitted to characteristics unique to this sample, limiting its external validity.

### Implications for interpretation

While this high R² demonstrates strong predictive relationships within our sample, readers should interpret these findings cautiously. The model’s exceptional explanatory power likely reflects the unique contextual conditions of Palestinian NICUs rather than universal relationships between AI awareness, experience, and worries. Future research in more diverse settings is essential to determine the generalizability of these relationships.

## Results

### Participants’ characteristics

The mean age of participants was 32.0 years (SD = 9.2), with 80.6% female. Most held a bachelor’s degree (50.7%), and mean NICU experience was 10.2 years (SD = 9.0). Only 14.5% (33 nurses) reported prior AI education, while 30.8% (70 nurses) had used AI in practice (Table 1).

**Table 1:** Demographic characteristics of the participants (N = 227)

Characteristics	N (%)	M(SD)
**Age**		32.0 (9.2)
**Gender**		
- Male	44 (19.4%)	
- Female	183 (80.6%)	
**Educational Level**		
- Diploma degree	59 (26.0%)	
- Bachelor’s degree	115 (50.7%)	
- Master’s degree and above	53 (23.3%)	
**Work experience in NICU**		10.2 (9.0)
**AI Education**		
- Yes	33 (14.5%)	
- No	194 (85.5%)	
**AI Use**		
- Yes	70 (30.8%)	
- No	157 (69.2%)	

### Perceived AI worries

Participants exhibited mid-range AI awareness scores (M = 2.7, SD = 0.5) and limited prior AI experience (M = 2.3, SD = 0.5). Total AI-related worries were moderate (M = 3.2, SD = 0.9), with the highest concerns related to healthcare providers (M = 3.4, SD = 1.0) and the lowest for regulatory/ethical issues (M = 3.0, SD = 0.7) (Table 2).

### Understanding low regulatory/ethical concerns

The relatively low concern for regulatory/ethical issues warrants comprehensive examination within the Palestinian healthcare context. This finding likely reflects multiple interconnected factors rather than simply indicating a lack of awareness:

#### Contextual factors

Several contextual factors may have shaped nurses’ perceptions and responses. Palestinian healthcare institutions currently lack comprehensive AI governance frameworks, limiting frontline nurses’ exposure to regulatory discussions. Without clear protocols or visible regulatory challenges, concerns about AI oversight may seem abstract or irrelevant. In resource-constrained settings, immediate clinical priorities, such as equipment shortages, staffing gaps, and basic patient safety, often take precedence. Regulatory issues related to emerging technologies may be viewed as a low priority. Cultural and organizational norms in Palestinian healthcare may also influence perceptions. A strong emphasis on collective decision-making and institutional trust could reduce individual concerns about regulatory oversight, especially when technology decisions are made at higher administrative levels.

#### Implications

The low level of concern about AI governance may signal a significant knowledge gap that needs urgent attention. As global AI adoption in healthcare accelerates, Palestinian institutions risk being unprepared for key ethical and legal challenges. These include algorithmic bias and fairness in clinical decision-making, ensuring data privacy and securing informed patient consent in AI-enhanced care, establishing accountability and liability when AI systems affect patient outcomes, and maintaining professional autonomy in environments where AI tools influence clinical decisions. Without targeted education and institutional preparedness, frontline staff may lack the awareness needed to navigate these issues.

This finding suggests an immediate need for targeted educational interventions to build ethical AI literacy among nursing staff before widespread implementation occurs.

**Table 2:** Distribution of AI awareness, experience, and worries (N = 227)

Variable	M	SD
AI Awareness	2.7	0.5
AI Previous Experience	2.3	0.5
Profession/Practice Worries	3.2	0.9
Healthcare Providers Worries	3.4	1.0
Data Management Worries	3.3	1.1
Regulatory/Ethics Worries	3.0	0.7
Total AI Worries	3.2	0.9

### Associations with AI worries

Spearman’s correlation revealed a weak positive association between education level and AI worries (ρ = 0.132, *p* = 0.048). This correlation, while statistically significant, represents a small effect size that warrants careful interpretation. The weak nature of this relationship suggests that educational background contributes modestly to AI-related concerns, potentially reflecting that higher education enhances critical thinking skills that enable nurses to anticipate implementation challenges, rather than indicating general technophobia among more educated nurses.

AI awareness (*r* = 0.821, *p* < 0.001) and AI experience (*r* = 0.272, *p* < 0.001) showed significant correlations with worries. Age, gender, work experience, AI education, and AI use were not significantly associated (Table 3).

**Table 3:** Correlations between AI worries and demographic/professional variables (N = 227)

Variable	*r*/ρ	*p*-value
Age	0.054	0.421
Gender*	-0.002	0.982
**Education Level**	**0.132**	**0.048**
Work Experience	0.089	0.183
AI Education	-0.001	0.994
AI Use	0.024	0.714
**AI Awareness**	**0.821**	**< 0.001**
**AI Experience**	**0.272**	**< 0.001**

*Gender analyzed via independent t-test (t = -0.02, *p* = 0.982).†Weak correlation (small effect size); may reflect enhanced critical thinking about implementation challenges rather than technophobia

### Predictors of AI worries

Multiple linear regression analysis identified three significant predictors of AI-related worries, explaining 84.6% of the variance (R² = 0.846). The model demonstrated strong predictive power while meeting all regression assumptions (Table 4).

**Table 4:** Predictors of AI worries: multiple linear regression

Predictor	B	β	*p*-value	95% CI	VIF
Education Level	0.074	0.060	0.026	0.009 to 0.140	1.25
AI Awareness	2.006	1.232	< 0.001	1.887 to 2.126	1.35
AI Previous Experience	-0.959	-0.580	< 0.001	-1.079 to -0.839	1.38

Note: B = unstandardized coefficient; β = standardized coefficient; CI = confidence interval; VIF = variance inflation factor. All VIF values were below 2.0, indicating no multicollinearity among predictors

## Discussion

This study, anchored in the Technology Acceptance Model (TAM), reveals that Palestinian NICU nurses’ perceptions of AI are shaped by a complex tension between awareness-driven critical appraisal and experience-driven acceptance. Our findings both support and extend TAM’s applicability to low-resource healthcare settings, demonstrating how the model’s core constructs manifest differently when technological exposure is limited, and structural constraints are significant.

### TAM applications in low-resource settings

The strong positive association between AI awareness and worries (*r* = 0.821, *p* < 0.001) challenges traditional TAM assumptions by suggesting that in resource-constrained environments, increased awareness does not automatically translate to acceptance. Instead, awareness may heighten concerns about implementation challenges, reliability issues during infrastructure failures, and potential workflow disruptions in settings where technological support is limited. This finding extends TAM by demonstrating that the relationship between awareness and acceptance is moderated by contextual factors such as infrastructure reliability and organizational support capacity.

Conversely, the inverse relationship between prior AI experience and worries (B = -0.959, *p* < 0.001) strongly supports TAM’s emphasis on ease of use as a catalyst for acceptance. This relationship suggests that hands-on experience with AI tools reduces abstract fears and builds confidence in technology capabilities, aligning with TAM’s proposition that direct interaction with technology influences adoption intentions [12].

### Comparative analysis with similar settings

To better contextualize our findings, comparison with studies from other low-resource settings reveals important patterns. Research from sub-Saharan Africa has shown similar relationships between limited technology exposure and heightened concerns about AI implementation, particularly regarding infrastructure reliability and maintenance capabilities. Studies from rural healthcare settings in Southeast Asia have reported comparable findings regarding the inverse relationship between hands-on experience and technology-related worries [13].

However, our findings contrast with research from urban settings in similar economic contexts, where nurses reported more balanced concern-to-acceptance ratios. This suggests that the conflict-affected nature of Palestinian healthcare may create unique implementation challenges beyond those typically encountered in other resource-constrained environments [14].

### Specific recommendations for implementation

Based on our findings, we propose specific, actionable recommendations:

### Low-cost AI tools for Palestinian nicus

Several low-resource AI solutions could offer practical support in Palestinian NICUs. Offline-capable vital sign monitoring systems can function during power outages and store data locally for later transmission, ensuring continuous monitoring despite infrastructure instability. Multilingual family education chatbots can deliver standardized responses to common questions in Arabic, easing the communication burden on nurses and improving information access for families. Predictive sepsis alert systems can analyze basic vital sign patterns to provide early warnings without requiring complex connectivity or maintenance. These solutions align with current constraints while addressing critical care and communication needs [15].

### NICU-specific simulation design

Targeted training can help nurses adapt to AI integration in resource-limited NICU settings. Scenario-based training modules using interactive simulations can prepare nurses for common NICU emergencies where AI tools offer decision support. Infrastructure failure protocols can train nurses to sustain AI-assisted care during power outages or connectivity disruptions, reinforcing system resilience. Cultural integration exercises can guide nurses in blending AI tools with traditional care practices and family-centered care, supporting ethical and culturally responsive implementation. These training approaches focus on practical readiness and contextual fit [16, 17].

### Regulatory/ethical concerns: a critical knowledge gap

The relatively low concern for regulatory/ethical issues (M = 3.0) represents a significant finding that requires targeted intervention. This gap suggests nurses may be unprepared for ethical dilemmas that accompany AI implementation [18]. Qualitative follow-up studies are urgently needed to explore whether this reflects genuine comfort with current ethical frameworks or indicates insufficient exposure to AI governance discussions.

## Strengths and limitations

### Strengths

This study pioneers’ insights into AI perceptions among neonatal intensive care unit (NICU) nurses in Palestine, offering a critical contribution to the underexplored literature on AI adoption in low-resource healthcare contexts, where infrastructural and sociopolitical barriers uniquely shape technological integration.

### Limitations

#### Methodological limitations

Several methodological limitations affect the interpretability and generalizability of the findings. The reliance on self-reported data introduces potential social desirability bias, especially in AI awareness items where participants may overstate their competence to appear technologically adept. The cross-sectional design captures perceptions at a single point in time, preventing analysis of how attitudes toward AI develop with increased exposure or experience. The study did not conduct confirmatory factor analysis due to sample size constraints, which limits validation of the WAAI-HCQ’s factor structure in this population and raises questions about construct validity in the Palestinian NICU context. Convenience sampling may have excluded key subgroups, such as nurses with low technological engagement, skewing the sample toward those more comfortable with AI and limiting broader applicability. Institutional restrictions also prevented access to non-responder data, making it impossible to assess how participants differed from those who declined, introducing further response bias and constraining the representativeness of the results.

#### Contextual limitations

The exceptionally high explained variance (R² = 84.6%) suggests potential unmeasured contextual variables, such as organizational culture, resource availability, political instability effects, or unique professional socialization patterns within Palestinian healthcare, that could significantly influence nurses’ worries and warrant dedicated investigation in future studies.

### Recommendations

#### For curriculum integration

Develop NICU-specific AI training modules to address ethics, data privacy, and clinical applications like neonatal sepsis prediction algorithms. These modules should bridge knowledge gaps and build nurse competencies. Include case-based learning grounded in Palestinian healthcare scenarios to ensure relevance. Provide hands-on simulations using low-cost AI tools designed to work with intermittent connectivity, reflecting local infrastructure challenges. Integrate ethics training that focuses on resource allocation dilemmas and cultural considerations, preparing nurses to navigate complex decisions in their context.

#### For policy development

Establish national AI task forces that include nurses, policymakers, and technologists to co-design guidelines tailored to the local context. These task forces should focus on creating culturally appropriate AI governance frameworks that address accountability and equity. They must develop implementation standards suited for resource-constrained settings to ensure practical and sustainable AI integration. Additionally, the task forces should establish nurse-led quality assurance protocols to monitor and evaluate AI tools, empowering nurses to maintain safety and effectiveness in clinical workflows.

## For technology implementation

Partner with NGOs and technology firms to deploy nurse-driven AI tools tailored for environments with intermittent connectivity. Ensure ongoing refinement by collecting regular feedback from nurses using the tools on the frontlines. Focus on pilot programs with strong evaluation frameworks to measure impact and identify areas for improvement. Use collaborative design processes that place nurse input at the center, ensuring tools fit real clinical workflows. Plan for sustainability by addressing long-term maintenance and ongoing training to support lasting adoption and effectiveness.

## Conclusion

This study highlights the nuanced perceptions of AI adoption among Palestinian NICU nurses, characterized by intermediate awareness levels and substantive concerns intertwined with limited practical experience. The findings underscore the dual role of education and hands-on exposure in shaping AI-related worries, as informed by TAM principles adapted to low-resource settings.

For Palestine and similar conflict-affected regions where AI integration is nascent, these insights offer critical guidance for stakeholders navigating the balance between technological advancement and workforce preparedness. The strong predictive relationships identified in our model, while requiring cautious interpretation due to their exceptional strength, provide a foundation for targeted interventions.

By addressing knowledge gaps through culturally appropriate education, developing context-sensitive policies, and fostering collaborative innovation partnerships, Palestinian healthcare systems can navigate AI adoption to enhance clinical outcomes while safeguarding professional roles and maintaining culturally appropriate care practices.

## Data Availability

No datasets were generated or analysed during the current study.
